# Effects of Dietary Isomaltooligosaccharide Levels on the Gut Microbiota, Immune Function of Sows, and the Diarrhea Rate of Their Offspring

**DOI:** 10.3389/fmicb.2020.588986

**Published:** 2021-01-08

**Authors:** Longlin Zhang, Xueling Gu, Jie Wang, Shuang Liao, Yehui Duan, Hao Li, Zehe Song, Xi He, Zhiyong Fan

**Affiliations:** ^1^College of Animal Science and Technology, Hunan Agricultural University, Changsha, China; ^2^Hunan Co-Innovation Center of Animal Production Safety, Hunan Agricultural University, Changsha, China; ^3^Hunan Provincial Key Laboratory of Animal Nutritional Physiology and Metabolic Process, Key Laboratory of Agro-ecological Processes in Subtropical Region, Institute of Subtropical Agriculture, Chinese Academy of Sciences, Hunan Provincial Engineering Research Center for Healthy Livestock and Poultry Production, Scientific Observing and Experimental Station of Animal Nutrition and Feed Science in South-Central, Ministry of Agriculture, National Engineering Laboratory for Pollution Control and Waste Utilization in Livestock and Poultry Production, Changsha, China

**Keywords:** reproductive performance, diarrhea, gut microbiota, sows, isomaltooligosaccharide

## Abstract

To investigate the effects of dietary isomaltooligosaccharide (IMO) levels on the gut microbiota, immune function of sows, and the diarrhea rate of their offspring, 120 multiparous gestating pig improvement company (PIC) sows with similar body conditions were selected and fed 1 of 6 diets: a basal diet with no supplement (control, CON), or a diet supplemented with 2.5 g/kg, 5.0 g/kg, 10.0 g/kg, 20.0 g/kg, or 40.0 g/kg IMO (IMO1, IMO2, IMO3, IMO4, or IMO5 group, respectively). Results showed that dietary treatments did not affect the reproductive performance and colostrum composition of sows (*P* > 0.05). However, compared to the CON, IMO reduced the diarrhea rate of suckling piglets (*P* < 0.05) and improved the concentrations of colostrum IgA, IgG, and IgM (*P* < 0.05). Moreover, IMO decreased the concentrations of serum D-lactate (D-LA) and lipopolysaccharides (LPS) at farrowing and day 18 of lactation (L18) (*P* < 0.05). High-throughput pyrosequencing of the 16S rRNA demonstrated that IMO shaped the composition of gut microbiota in different reproductive stages (day 107 of gestation, G107; day 10 of lactation, L10) (*P* < 0.05). At the genus level, the relative abundance of g_*Parabacteroides* and g_*Slackia* in G107 and g_*Unclassified_Peptostreptococcaceae*, g_*Turicibacter*, g_*Sarcina*, and g_*Coprococcus* in L10 was increased in IMO groups but the g_*YRC22* in G107 was decreased in IMO groups relative to the CON group (*P* < 0.05). Furthermore, the serum D-LA and LPS were negatively correlated with the genus g_*Akkermansia* and g_*Parabacteroides* but positively correlated with the genus g_*YRC22* and g_*Unclassified_Peptostreptococcaceae*. Additionally, the colostrum IgA, IgG, and IgM of sows were positively correlated with the genus g_*Parabacteroides*, g_*Sarcina*, and g_*Coprococcus* but negatively correlated with the genus g_*YRC22*. These findings indicated that IMO could promote the immune activation and had a significant influence in sows’ gut microbiota during perinatal period, which may reduce the diarrhea rate of their offspring.

## Introduction

The perinatal period of sows is the transition stage from gestation to lactation, which generally refers to the combined period of late gestation and early lactation (10 days before after delivery). And sows are the core of modern large-scale pig production; their health and reproductive performance are the key to the economic benefits of pig farms. There were massive researches which suggest that the gut microbiota homeostasis of sow is important for the healthy gut development of their offspring during perinatal period (Zambrano et al., 2016; Grześkowiak et al., 2020). In addition, the physiological condition of suckling piglets is closely connected with the sows in the first weeks of life, including energy and nutrient supply (Klobasa et al., 1987; Gao et al., 2020), immunological protection (Salmon et al., 2009) and the microbial colonization of the gastrointestinal tract. So far, there are increasingly studies on the sow-piglet-axis, but there are not many systematic research literatures in this field.

Previous studies have demonstrated that a probiotic treatment of sows altered the composition of the gut microbiota in their offspring (Mori et al., 2011; Baker et al., 2013; Starke et al., 2013). Isomaltooligosaccharides (IMO), as a functional oligosaccharide, is considered to act as a prebiotic, since it can modulate the composition and metabolic activity of the gut microbiota, which might potentially enhance the health of the host organism (Ketabi et al., 2011). In particular, previous studies have shown that *Bifidobacterium* and *Lactobacillus* were increased in fecal microbiota, when different doses of IMO were supplemented to the diets (Ketabi et al., 2011; Yen et al., 2011; Likotrafiti et al., 2014). Besides, IMO is also known for its potential to activate the immune system and to enhance the host’s resistance to diseases and oxidation (Wu et al., 2017). However, no studies have been conducted to evaluate the effects of dietary IMO in gestating and lactating sows, although dietary IMO supplementation directly improved immune status and diarrhea of piglets (Wang et al., 2016; Wu et al., 2017). This aspect might be interesting, as a modulation of the gut microbiota of the sows might also influence the structure and composition of bacteria in the intestinal tract of their piglets. In addition, few studies have evaluated the potential of prebiotics in the sow-piglet-axis up to now, particular IMO. Therefore, it is necessary to study on the mechanism of IMO supplementation with respect to sow-piglet-axis.

Consequently, the purpose of this study was to study the effects of dietary IMO levels on reproductive performance, colostrum composition, immune index and gut microbiota of sows and diarrhea of offspring.

## Materials and Methods

### Animal, Diets and Experimental Design

The protocol of this study was approved by the Institution Animal Care and Use Committee of college of Animal Science and Technology, Hunan Agricultural University (Changsha, China), and was conducted in accordance with the National Institutes of Health (Changsha, China) guidelines for the care and use of experimental animals. The IMO (IMO-900; purity ≥ 90%, with total isomaltose, panose, and isomaltotriose contents > 45%) was provided by the Baolingbao Biology Company (Shandong, China).

One hundred and twenty late pregnant sows (day 85 of gestation, PIC) with an initial body weight of 253.36 ± 14.30 kg and parity of 5.27 ± 1.58 were randomly allocated to 1 of 6 dietary treatments with 20 replicates based on body weight, parity and back fat. The treatments are: 1) a basal diet from late gestation to farrowing (CON group), 2) a basal diet plus 2.5g/kg, 5.0g/kg, 10.0g/kg, 20.0g/kg or 40.0g/kg IMO (IMO1, IMO2, IMO3, IMO4, or IMO5 groups). The composition of basal diets (Supplementary Table 1) was formulated in compliance with NRC (1998) nutrient requirements.

Sows were housed in 2.0 m × 0.6 m concrete-floored farrowing pen during day 85 to day 107 of gestation. The average amount fed to sows in each group was half provided at each feeding for a total of 2.6–2.8 kg, twice a day (08:00 am and 15:00 pm). During gestation day 108 to lactation, the sows were housed indoors in 2.13 m × 0.66 m concrete-floored delivery room pen. The average amount fed to sows in each group was half provided at each feeding for a total of 3.2 kg, twice a day (08:00 am and 16:30 pm). Before farrowing days 1–2, the average feeding amount dropped to 2.0 kg d^–1^. On the farrowing day (day 0 of lactation), the sows initially received a total of 1.0 kg day^–1^ of their lactation diets, which was then increased by 0.8 kg day^–1^ on days 1 and 2 and by 1.0 kg day^–1^ on days 3 and 4 until arriving at their maximum feed intake. To feed sows diets *ad libitum* to ensure that the sow’s trough has surplus fodder from day 5 of lactation to weaning. Sows were provided *ad libitum* access to water during the experimental period. The experiment was carried out in Hunan Xinguangan Agriculture and Animal Husbandry Co., Ltd. Pingjiang Branch (Xinguangan, Inc., Hunan, China), and the feeding management and immunization procedure were carried out in accordance with the company’s standard of breeding management. The trial lasted for 60 days.

### Sample Collection

Seven sows per group were randomly selected for sample collection. Fresh feces were collected directly by massaging the rectum of sow on G107 and L10. Then, 60 samples stored in dry ice were transported to the laboratory and then stored at -80°C until analysis. A 10-mL blood sample of sow from the ear vein was collected on farrowing day within 2 h after delivery and L18 after an overnight fasting period of 16–18 h. Serum samples were obtained by centrifuging at 3,000 × *g* for 15 min at 4°C after standing for 1 h at 4°C. Then the samples were immediately stored at −80°C for the next analysis. Within two hours after farrowing, seven sows in each group were randomly selected for milk sample collection by hand-milking of four to six teats.

### Reproductive Performance and Diarrhea Rate of Piglets

The total number of born, born alive, born robust (weight greater than 800 g), stillborn, and mummy number was recorded, so were average piglet birth weight (BW) at farrowing and lactation as well as average daily feed intake (ADFI) during lactation. On this basis, the survival rate of piglets, litter weight gain (from day 3 after birth to weaning) and total milk yield [(weaning litter weight-initial litter weight)/4] were calculated. In addition, from birth to weaning, the fecal score of piglets of each litter was recorded daily and their diarrhea rate during days 1–3, 1–7, 1–14, and 1–21 after birth were also be calculated.

### Sow’s Milk Composition and Immunity, and Serum D-Lactate and Lipopolysaccharides

The milk samples of sows in each group were separately analyzed for the concentrations of fat, protein, lactose, urea nitrogen, fatting dry matter, and total dry matter using a Milko-Scan FT 120 (Foss Electric, Hillerford, Denmark). Colostrum and serum concentrations of immunoglobulin G (IgG), immunoglobulin A (IgA), and immunoglobulin M (IgM) were determined by the radial immunodiffusion method using a commercial kit (Wuhan Biological Engineering Co., Ltd, Wuhan, China). Besides, the serum D-lactate (D-LA) and lipopolysaccharides (LPS) were assessed by ELISA using commercially available kits (Wuhan Biological Engineering Co., Ltd, Wuhan, China).

### DNA Extraction 16S rDNA Amplification and 16S rRNA Sequencing

DNA was extracted from fecal samples of sows (G107 and L10) using a Stool DNA Isolation Kit (Tiangen Biotech Co., Ltd., Beijing, China). The V3–V4 hypervariable region of the bacterial 16S rRNA gene was amplified using universal primers (338F and 806R). For each fecal sample, a 10-digit barcode sequence was added to the 5’ end of the forward and reverse primers (provided by Allwegene Company, Beijing, China). The PCR components contained 5 μL of Q5 reaction buffer (5×), 5 μL of Q5 High-Fidelity GC buffer (5×), 0.25 μL of Q5 high-fidelity DNA polymerase (5 U/μL), 2 μL (2.5 mM) of dNTPs, 1 μL (10 uM) of each forward and reverse primer, 1 μL of DNA template, and 9.75 μL of ddH_2_O. Cycling parameters were 98°C for 5 min, followed by 25 cycles at 98°C for 30 s, 52°C for 30 s, and 72°C for 1 min, and a final extension at 72°C for 5 min. PCR amplicons were purified with Agencourt AMPure beads (Beckman Coulter, Indianapolis, IN, United States) and quantified using the PicoGreen dsDNA Assay Kit (Invitrogen, Carlsbad, CA, United States). After the individual quantification step, amplicons were pooled in equal amounts, and pair-end 2 × 300 bp sequencing was performed using the Illlumina MiSeq platform with MiSeq Reagent Kit v3 at Shanghai Personal Biotechnology Co., Ltd (Shanghai, China). The sequences were clustered into operational taxonomic units (OTUs) at a similarity level of 97% to generate rarefaction curves and to calculate the richness and diversity indices. OTUs representing <0.005% of the population were removed and taxonomy was assigned by the Ribosomal Database Project (RDP) classifier.

The Qiime^1^ software (Caporaso et al., 2010) was employed to process the sequencing data and perform cluster analysis, ACE abundance indexing, and Simpson diversity indexing of the analysis results. In addition, USEARCH^2^ was used to exclude chimeric sequences. Beta diversity analysis was used to investigate the structural variation of microbial communities across samples using UniFrac distance metrics. The Spearman’s rho nonparametric correlations between the gut microbiota and immune-related indexes were determined using R packages (v3.5.2).

### Statistical Analysis

An individual sow served as the experimental unit. All statistical analyses were performed using SPSS 20.0 software (SPSS Inc., Chicago, IL, United States). The differences among groups were compared using covariance analysis, one-way ANOVA and Duncan multiple range test. In addition, the reproductive performance and colostrum composition of sows were compared with use of independent-samples *T*-test, and the diarrhea rates of suckling piglets were compared with use of chi-square analysis. Significance was set at *P* < 0.05.

## Results

### Reproductive Performance on Sows

The effects of dietary IMO levels on reproductive performance of sows were shown in Table 1. There were also no effects of IMO treatment on mainly reproductive performance of sows (*P* > 0.05), in addition to mummy number and lactation ADFI (*P* < 0.05).

**TABLE 1:** Effects of different doses of IMO on the reproductive performance of sows.

Items	CON	IMO^a^					*P*-value				
		2.5 g/kg	5.0 g/kg	10.0 g/kg	20.0 g/kg	40.0 g/kg	IMO1/CON	IMO2/CON	IMO3/CON	IMO4/CON	IMO5/CON
Total pigs born, *n*	12.68 ± 0.89	11.52 ± 0.60	13.00 ± 0.58	12.35 ± 0.51	12.61 ± 0.63	12.52 ± 0.52	0.084	0.577	0.216	0.695	0.087
Pigs born alive, *n*	11.58 ± 0.86	11.00 ± 0.55	11.94 ± 0.59	11.94 ± 0.52	11.80 ± 0.64	11.85 ± 0.53	0.085	0.290	0.561	0.712	0.120
Pigs born robust, *n*	11.26 ± 0.82	10.74 ± 0.56	11.46 ± 0.60	11.40 ± 0.52	11.51 ± 0.64	11.61 ± 0.54	0.254	0.530	0.659	0.205	0.200
Stillbirth number, *n*	1.08 ± 0.25	0.50 ± 0.22	1.07 ± 0.27	0.41 ± 0.24	0.80 ± 0.29	0.68 ± 0.24	0.553	0.147	0.410	0.139	0.494
Mummy number, *n*	0.15 ± 0.14	0.26 ± 0.13	0.22 ± 0.16	0.21 ± 0.14	0.20 ± 0.17	0.12 ± 0.14	0.037	0.124	0.049	0.012	0.917
Average piglet BW, kg	1.43 ± 0.05	1.43 ± 0.04	1.49 ± 0.05	1.44 ± 0.05	1.53 ± 0.06	1.42 ± 0.05	0.140	0.060	0.315	0.156	0.606
Survival rate,	0.95 ± 0.02	0.94 ± 0.02	0.97 ± 0.02	0.97 ± 0.02	0.95 ± 0.02	0.96 ± 0.02	0.722	0.124	0.120	0.306	0.613
Litter weight gain, kg	32.90 ± 2.03	35.04 ± 1.90	34.34 ± 2.04	34.91 ± 2.00	33.85 ± 2.20	31.71 ± 2.24	0.295	0.978	0.922	0.816	0.719
Lactation ADFI, kg	5.97 ± 0.15	5.90 ± 0.14	6.04 ± 0.15	5.95 ± 0.15	6.04 ± 0.16	5.87 ± 0.16	0.011	0.154	0.055	0.346	0.013

*The results were expressed as mean ± SEM. ^*a*^IMO: IMO1, 2.5 g/kg IMO group; IMO2, 5.0 g/kg IMO group; IMO3, 10.0 g/kg IMO group, IMO4, 20.0 g/kg IMO group, IMO5, 40.0 g/kg IMO group. BW, birth weight; ADFI, average daily feed intake.*

### The Diarrhea Rate of Suckling Piglets

**FIGURE 1:**
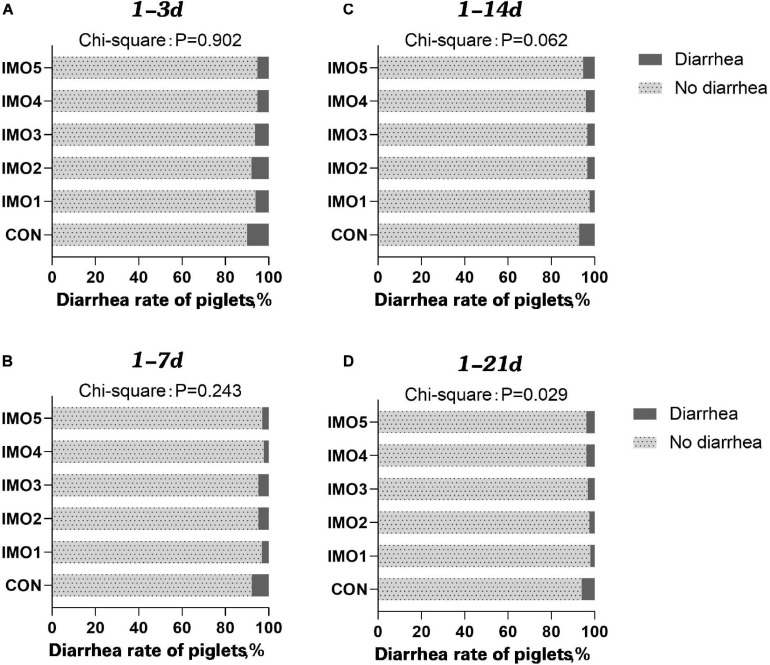
Effects of different doses of IMO on the diarrhea of suckling piglets in a different stage [**(A)**, 1–3 days; **(B)** 1–7 days; **(C)** 1–14 days; **(D)** 1–21 days]. Data were compared with use of chi-square analysis (*P* < 0.10).

The results of dietary IMO levels on diarrhea rate of piglets were shown in Figure 1. The diarrhea rate of piglets was awfully high at days 1 to 14 of lactation (1–14 days) and days 1 to 21 of lactation (1–21 days), whereas dietary IMO reversed the diarrhea rate of piglets in a dose-dependent manner (*P* < 0.05, Figures 1C,D).

### The Colostrum Composition and Immunity of Sows

**FIGURE 2:**
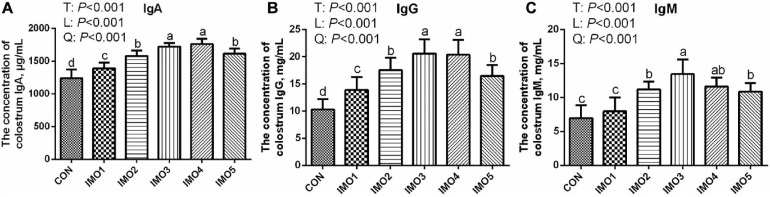
Effects of dietary isomaltooligosaccharide (IMO) levels on the concentrations of colostrum IgA **(A)**, IgG **(B)**, and IgM **(C)**. Data are presented as means ± SD (*n* = 7). (a–d) Significant effects of treatment (*P* < 0.05; values with different lowercase letters are significantly different; T, total; L, linear; Q, quadratic).

Effects of dietary IMO levels on colostrum composition and immunity were shown in Table 2 and Figure 2. The results displayed that dietary IMO had no marked effect on the colostrum composition of sows (*P* > 0.05, Table 2). However, sows fed the IMO had a higher IgA, IgG, and IgM concentration in colostrum, with the highest values observed in IMO3 and IMO4 groups (*P* < 0.001, Figure 2).

**TABLE 2:** Effects of different doses of IMO on colostrum composition of sows.

Items	CON	IMO^a^					*P*-value				
		2.5 g/kg	5.0 g/kg	10.0 g/kg	20.0 g/kg	40.0 g/kg	IMO1/CON	IMO2/CON	IMO3/CON	IMO4/CON	IMO5/CON
Milk fat, %	0.92 ± 0.07	0.88 ± 0.10	0.79 ± 0.08	0.78 ± 0.05	0.77 ± 0.06	0.81 ± 0.12	0.601	0.660	0.382	0.700	0.279
Milk protein, %	2.99 ± 0.19	2.65 ± 0.22	3.06 ± 0.10	2.98 ± 0.13	3.05 ± 0.15	2.82 ± 0.19	0.228	0.171	0.303	0.676	0.798
Milk lactose, %	0.67 ± 0.02	0.69 ± 0.02	0.69 ± 0.02	0.69 ± 0.03	0.65 ± 0.02	0.70 ± 0.02	0.684	0.915	0.712	0.911	0.918
Milk urea nitrogen, mg⋅dL^–1^	10.70 ± 0.28	9.72 ± 0.85	9.58 ± 0.68	9.38 ± 0.39	10.15 ± 0.53	9.10 ± 0.74	0.296	0.515	0.320	0.772	0.749
Milk total dry matter, %	5.93 ± 0.20	5.62 ± 0.26	5.89 ± 0.14	5.80 ± 0.12	5.81 ± 0.17	5.72 ± 0.26	0.192	0.348	0.282	0.077	0.832
Milk somatic cell count, ml^–1^	135.50 ± 15.4	173.50 ± 25.8	139.00 ± 25.8	162.00 ± 27.7	128.00 ± 2.74	179.00 ± 15.98	0.060	0.067	0.704	0.216	0.084

*The results were expressed as mean ± SEM. ^*a*^IMO: IMO1, 2.5 g/kg IMO group; IMO2, 5.0 g/kg IMO group; IMO3, 10.0 g/kg IMO group, IMO4, 20.0 g/kg IMO group, IMO5, 40.0 g/kg IMO group.*

### The Serum Immunity of Sows

**FIGURE 3:**
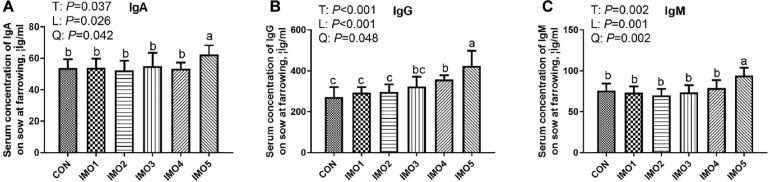
Effects of dietary isomaltooligosaccharide (IMO) levels on the concentrations of serum IgA **(A)**, IgG **(B)**, and IgM **(C)**. Data are presented as means ± SD (*n* = 7). (a–c) Significant effect of treatment (*P* < 0.05; values with different lowercase letters are significantly different; T, total; L, linear; Q, quadratic).

As shown in Figure 3, sows in the IMO5 group exhibited the highest concentrations of serum IgA, IgG, and IgM (*P* < 0.05) at farrowing. Besides, the IMO4 significantly increased the serum IgG concentration compared with CON, IMO1, and IMO2 groups (*P* < 0.05, Figure 3B).

### The Serum Biomarker of Sows

**FIGURE 4:**
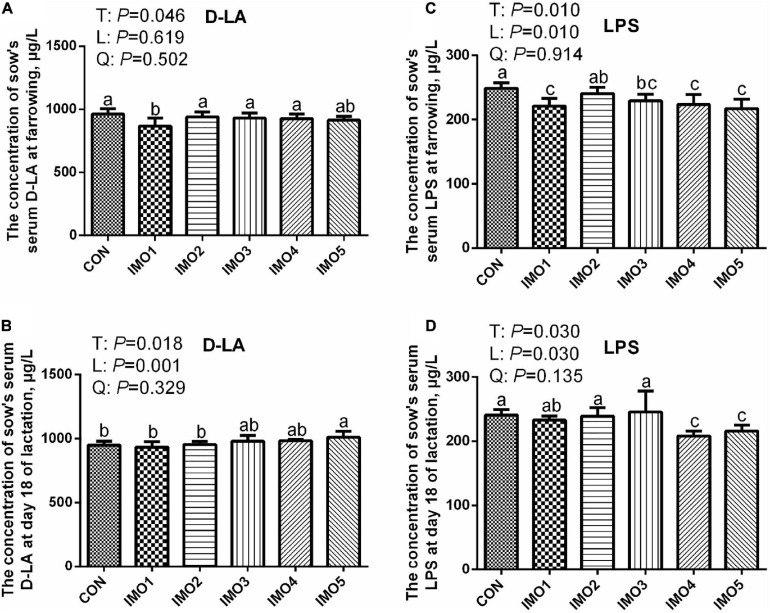
Effects of dietary isomaltooligosaccharide (IMO) levels on the concentrations of sows’ serum D-LA **(A)** and LPS **(C)** at farrowing as well as serum D-LA **(B)** and LPS **(D)** at day 18 of lactation. Data are presented as means ± SD (*n* = 7). (a–c) Significant effect of treatment (*P* < 0.05; values with different lowercase letters are significantly different; T, total; L, linear; Q, quadratic).

At farrowing, the D-LA concentration in IMO1 group and LPS concentration in IMO1, IMO3, IMO4 and IMO5 groups were lower than those in the CON group (Linear, *P* < 0.05, Figures 4A,C). In addition, sows fed the diets containing IMO5 had a higher concentration of serum D-LA compared to IMO1 and IMO2 in L18 (Linear, *P* < 0.05), but the lower concentration of serum LPS was shown in IMO4 and IMO5 groups (Linear, *P* < 0.05, Figures 4B,D).

### The Gut Microbiota Diversity and Composition of Sows

**FIGURE 5:**
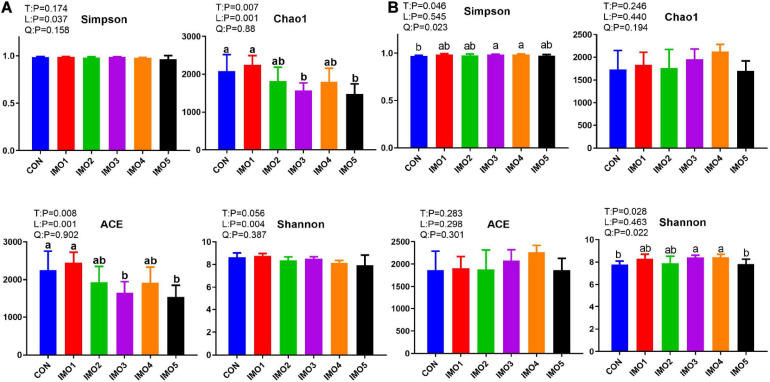
Effects of dietary isomaltooligosaccharide (IMO) levels on the α-diversity of gut microbiota at day 107 of gestation **(A)** and at day 10 of lactation **(B)** of sows. Simpson index, Chao1 index, ACE index, and Shannon index. Data are presented as means ± SD (*n* = 5). (a–c) Significant effect of treatment (*P* < 0.05; values with different lowercase letters are significantly different; T, total; L, linear; Q, quadratic).

Isomaltooligosaccharide changed the gut microbiota diversity of sows in G107 and L10 but the trend was different. The Chao 1 and ACE diversity indices were significantly reduced by dietary IMO supplementation in sows (expect IMO1 group) compared with the CON group in G107 (Linear, *P* < 0.05, Figure 5A). The Simpson and Shannon diversity indices were increased in all IMO groups, and the significantly difference was observed among the CON group and IMO3 and IMO4 groups in L10 (Quadratic, *P* < 0.05, Figure 5B).

**FIGURE 6:**
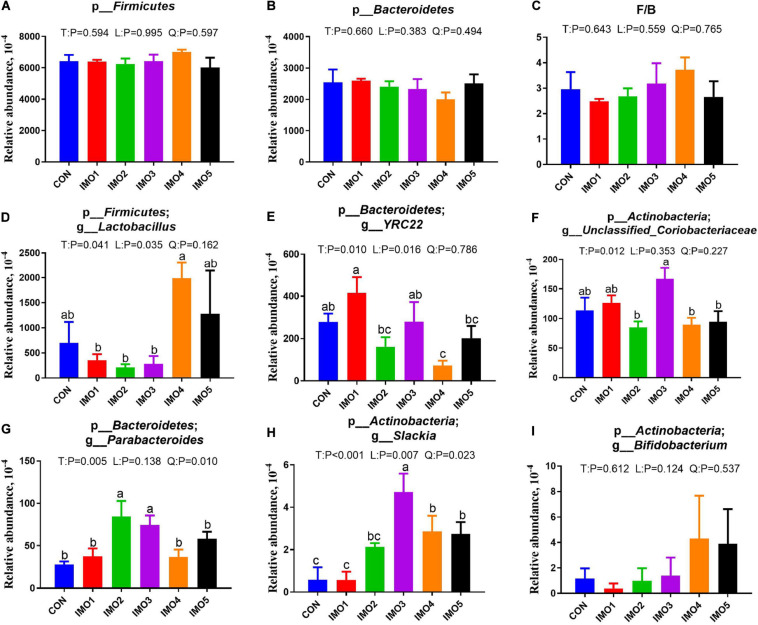
Effects of dietary isomaltooligosaccharide (IMO) levels on the gut microbiota compositions at the phylum level in sows. **(A)** p_*Firmicutes*, **(B)** p_*Bacteroidetes*, **(C)** F/B, *Firmicutes*/*Bacteroidetes*, **(D)** p_*Firmicutes*; g_*Lactobacillus*, **(E)** p_*Bacteroidetes*;g_*YRC22*, **(F)** p_*Actinobacteria*; g_*Unclassified_Coriobacteriaceae*, **(G)** p_*Bacteroidetes*; g_*Parabacteroides*, **(H)** p_*Actinobacteria*; g_*Slackia*, **(I)** p_*Actinobacteria*; g_*Bifidobacterium*. Data are presented as means ± SEM (*n* = 5). (a–c) Significant effect of treatment (*P* < 0.05; values with different lowercase letters are significantly different; T, total; L, linear; Q, quadratic).

**FIGURE 7:**
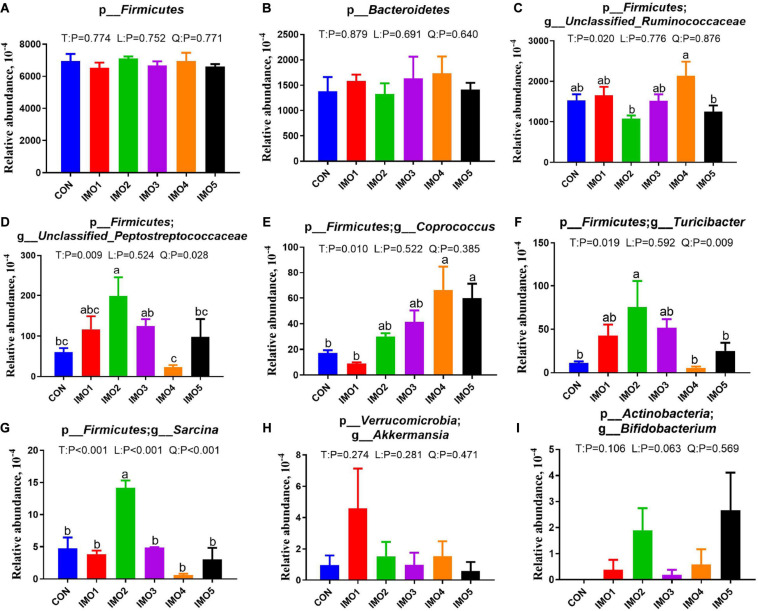
Two major bacterial phyla and the changes in the seven distinct genera in gut microbiota composition of sows at day 10 of lactation. **(A)** p_*Firmicutes*, **(B)** p_*Bacteroidetes*, **(C)** p_*Firmicutes*; g_*Unclassified_Ruminococcaceae*, **(D)** p_*Firmicutes*; g_*Unclassified_Peptostreptococcaceae*, **(E)** p_*Firmicutes*; g_*Coprococcus*, **(F)** p_*Firmicutes*; g_*Turicibacter*, **(G)** p_*Firmicutes*; g_*Sarcina*, **(H)** p_*Verrucomicrobia*; g_*Akkermansia*, **(I)** p_*Actinobacteria*; g_*Bifidobacterium*. Data are presented as means ± SEM (*n* = 5). (a–c) Significant effect of treatment (*P* < 0.05; values with different lowercase letters are significantly different; T, total; L, linear; Q, quadratic).

All 60 fecal samples were subjected to 16S rRNA gene sequencing. Illumina Miseq sequencing of the V3-V4 regions of bacterial 16S rRNA genes generated 1,094,499 and 1,132,136 high-quality sequences in G107 and L10, respectively (Supplementary Table 2). On the basis of 97% sequence similarity, we obtained 7,806 and 7,969 OTUs in G107 and L10, respectively. Further, variations in the microbial composition of all groups were explored. LEfSe analysis of the bacterial community was used to filter the significantly different OTUs among groups and the results showed that there exist dramatic differences in microbial composition between the treatment groups and the CON group (Supplementary Figure 1). There were no significant difference in the relative abundance of bacterial p_*Firmicutes* and p_*Bacteroidet*es and its ratio (F/B) among the all treatment groups in G107 and L10 (*P* > 0.05, Figures 6A–C, 7A,B). g_*Lactobacillus* increased but g_*YRC22* showed a remarkable reduction in IMO4 group compared with the CON group in G107 (Linear, *P* < 0.05, Figures 6D,E). Sows in IMO3 group had a highest relative abundance of g_*Unclassified_Coriobacteriaceae* and g_*Slackia*, and it significantly differ from IMO2, IMO4, and IMO5 groups in the relative abundance of g_*Unclassified_Coriobacteriaceae* and CON group, IMO1,IMO2, IMO4, and IMO5 groups in the relative abundance of g_*Slackia* (Total, *P* < 0.05, Figures 6F,H). Besides, the relative abundance of g_*Parabacteroides* was enhanced in IMO2 and IMO3 groups (Quadratic, *P* < 0.05, Figure 6G) and the relative abundance of g_*Bifidobacterium* was increased in IMO4 and IMO5 groups (*P* > 0.05, Figure 6I). At the genus level, the relative abundances of g_*Unclassified_Ruminococcaceae* and g_*Coprococcus* were the highest (Total, *P* < 0.05, Figures 7C,E) but the g_*Unclassified_Peptostreptococcaceae* and g_*Sarcina* was the lowest in IMO4 group in L10 (*P* < 0.05, Figures 7D,G). In addition, the relative abundances of g_*Unclassified_Peptostreptococcaceae*, g_*Turicibacter* and g_*Sarcina* were the highest in IMO2 group (Quadratic, *P* < 0.05, Figures 7D,F,G) and the relative abundance of g_*Akkermansia* was the highest in IMO1 group (*P* > 0.05, Figure 7H). Furthermore, IMO increased the relative abundance of g_*Bifidobacterium* compared with the CON (*P* > 0.05, Figure 7I). Therefore, the above results indicated that the gut microbiota composition of sows was profoundly altered during late pregnancy and lactation.

### Correlations Between Gut Microbiota and Serum Biomarker and Colostric Immunoglobulin

**FIGURE 8:**
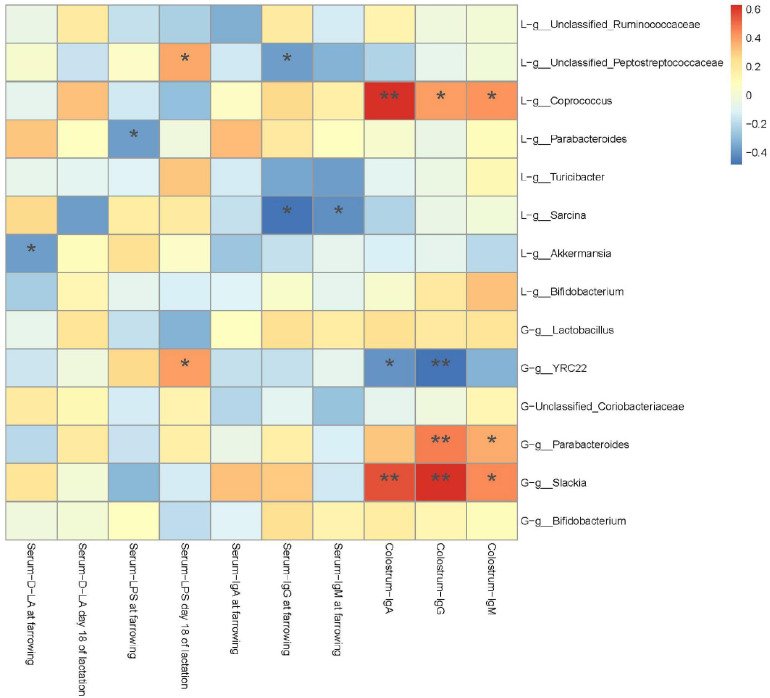
Heatmap of the Spearman’s *r* correlations between the gut microbiota significantly modified by different dietary treatments and period of sows. Data are presented as means ± SEM (*n* = 5 or 7). *P < 0.05; **P < 0.01 (following the Spearman’s correlation analysis). G, at day 107 of gestation; L, at day 10 of lactation.

A Spearman’s correlation analysis was performed to evaluate the potential link between alterations in gut microbiota composition in G107 and L10 and serum D-LA, LPS, IgA, IgG, and IgM of sows at farrowing and L18 or IgA, IgG, and IgM in colostrum of sows at farrowing (Figure 8). The serum D-LA and LPS at farrowing was negatively correlated with the genus g_*Akkermansia* and g_*Parabacteroides* in L10 (*P* < 0.05), respectively. However, the serum LPS in L18 was positively correlated with the genus g_*YRC22* in G107 and the genus g_*Unclassified_Peptostreptococcaceae* in L10 (*P* < 0.05). Moreover, the IgA, IgG, and IgM in colostrum of sows at farrowing was positively correlated with the genus g_*Parabacteroides* and g_*Slackia* in G107 and g_*Coprococcus* in L10 but negatively correlated with the genus g_*YRC22* in G107 (*P* < 0.05).

## Discussion

The health of sow plays a crucial role in the pig production, and sows’ reproductive performance is an important index of economic benefit of pig farm. However, sows in the late gestation due to rapid fetal growth caused by insulin resistance and the body’s metabolic syndrome will lead to gut microbiota disorder, which will affect the reproductive performance of the sows and the growth and health of their offspring (Tan et al., 2016; Cheng et al., 2020). IMO is one of the oligosaccharides which produced by enzymatic conversion of starch and widely used in the food and feed industry, which has a wide spectrum of biological activities (Ketabi et al., 2011). Previous studies have shown that dietary oligosaccharides supplementation in sows improves sows’ performance by regulating the homeostasis of gut microbiota (Li et al., 2012; Duan et al., 2019). Contrary to these results, our current study showed that IMO supplementation exerted no effects on the mainly reproductive performance of sows. This discrepancy could be attributed to the time phase of IMO supplementation and different polymerization degree of commercial IMO (Hu et al., 2013).

Interestingly, we found that dietary IMO supplementation in sows significantly reduced the diarrhea rate of piglets during lactation, especially for low dose. Consistently, Wang et al. (2016) reported that the diarrhea rate of piglets linearly declined as the IMO level increased, and the beneficial effects may be due to enhanced immune status of pigs. During lactation, milk is one of the most important ways that the sows connect with its offspring (Wu et al., 2020). Moreover, the breast milk is the only source of the energy and immunity of the piglets (Xie et al., 2015), which contributes to immune system maturation, organ development, and healthy microbial colonization (Parigi et al., 2015). Many studies have showed that the milk composition and its immuneglobulin concentration play key roles in the healthy growth and development of piglets (Lonnroth et al., 1988; Charneca et al., 2015), which is closely related to the gut microbiota (Solís et al., 2010; Burton et al., 2017) and may affect the growth and development of the piglets (Kirmiz et al., 2018). A recent study indicated that chitooligosaccharides supplementation modified milk composition (Cheng et al., 2015). Similarly, previous studies have also shown that the sows fed mannanoligosaccharides improved the colostrum and milk IgA IgG, IgM, and the serum IgG level in the suckling piglets (Newman and Newman, 2001). Especially during early lactation, the diarrhea of piglets occurred due to the colostrum immunoglobulin. Therefore, we measured the colostrum composition and immunoglobulin (IgA, IgG, and IgM) of sows. We found that IMO supplementation did not affect colostrum composition of sows, but improved the concentrations of colostrum IgA, IgG, and IgM, which may explain why the diarrhea rate of piglets decreased.

In addition, the injury of gut barrier function is also closely related to the milk quality. Perez et al. (2007) found that intestinally derived bacterial components are transported to the lactating breast within mononuclear cells, which affected the milk quality and the offspring health. Bacterial translocation usually caused by the destruction of gut barrier (MacFie, 2000). The concentrations of serum LPS and D-LA can be used to give expression to the gut barrier function, and if its concentration improved in serum, the gut barrier have been destroyed (Xu et al., 2018). In the present study, the diets of sows containing IMO especially at low and high doses have lower concentrations of serum LPS and D-LA at farrowing and L18, which reflected that the function of IMO protected the intestinal barriers.

The gut microbiota is one of the most important intestinal barriers, whose homeostasis will also affect the body immunity, including the milk immunity (Rooks and Garrett, 2016). As we know, the level of immunoglobulin expression in serum of sows can immediately reflect the body immunity, which will also cause the changes of gut bacteria. In the present study, we found that IMO with different doses changed the concentrations of serum IgA, IgG, and IgM, especially IMO5 was very significant increased the immunoglobulin expression in serum of sows. To some extent, this suggests that IMO can increase the immunity of perinatal sows, which influence the structure of gut microbiota in sows. Furthermore, the establishment of gut microbiota of piglets was influenced by sow’s gut microbiota, which has already been demonstrated after a probiotic treatment of mother sows (Mori et al., 2011; Baker et al., 2013; Starke et al., 2013). Recent studies have suggested that diarrhea is strongly related to the dysbiosis of gut microbiota (Shrivastava et al., 2017; The et al., 2018). In addition, the disorderliness of gut microbiota during perinatal period of sows will affect its milk quality and gut microbiota of piglets, which may lead to diarrhea in piglets. Therefore, the functional pro(e)biotic supplementation to the diets of sows during perinatal period may improve the diarrhea of piglets by regulating sow’s gut microbiota.

In the present study, we found that IMO with different doses changed the composition and structure of the gut microbiota of perinatal sows, especially G107 and L10. Except for the numbers of g_*YRC22*, the bacterial groups in the feces of the sows were increased with varying degrees by the dietary inclusion of IMO. In G107, dietary supplementation of IMO increased the abundance of g_*Parabacteroides*, belonging to p_*Bacteroidetes*, which were comprises anti-inflammatory bacteria (Ishioka et al., 2017). The relative abundance of bacterial g_*Slackia*, belonging to p_*Actinobacteria*, was also increased. It can produce some bioactive substances to play a protective role and correlated with immunity. The same as our result, g_*Parabacteroides* and g_*Slackia* were closely correlated with colostrum IgA, IgG, and IgM, which may influence the diarrhea of piglets by this way.

Moreover, dietary supplementation of IMO in L10 can also significantly enhanced the relative abundance of bacterial g_*Unclassified_Peptostreptococcaceae*, g_*Coprococcus*, g_*Turicibacter* and g_*Sarcina*, which both belong to the p_*Firmicutes*. g_*Unclassified_Peptostreptococcaceae* was the dominant bacteria genus to produce the volatile fatty acids (VFAs), which contributed to the energy metabolism of host (Li et al., 2018). g_*Coprococcus* have been found to play beneficial roles in maintaining intestinal stability, also reported as the butyrate-producing genus (Nishino et al., 2018). g_*Turicibacter* and g_*Sarcina* are also in connection with intestinal barrier function and inflammation (Liu et al., 2014; Le Sciellour et al., 2019). It systematically enhancing effect of dietary IMO on the numbers of beneficial bacterium in sows can be assumed.

## Conclusion

In conclusion, IMO could reduce the diarrhea rate of their offspring, the effects might be attributed to the enhanced immune system of piglets. This suggests that the reduced rate of diarrhea in piglets is mainly because of the role of immunoglobulin in the sows’ milk, although the IMO still had a significant influence in sows’ gut microbiota, which was significantly correlated with immunoglobulin in milk. In our studies, it has evaluated the potential of IMO with regard to the sow-piglet-axis, but the concrete mechanism needs to be further researched.

## Data Availability Statement

The original contributions presented in the study are publicly available. This data can be found here: https://www.ncbi.nlm.nih.gov/bioproject/PRJNA681971.

## Ethics Statement

The animal study was reviewed and approved by the National Institutes of Health (Changsha, China) guidelines for the care and use of experimental animals.

## Author Contributions

ZYF, ZHS, and XH conceived and designed the experiment. LLZ, XLG, JW, and SL performed the experiments. LLZ, XLG, and HL analyzed the data and wrote the manuscript. YHD and ZYF revised the manuscript. All authors contributed to the article and approved the submitted version.

## Conflict of Interest

The authors declare that the research was conducted in the absence of any commercial or financial relationships that could be construed as a potential conflict of interest. The reviewer XX declared a shared affiliation with one of the authors YHD, to the handling editor at the time of the review.

## Footnotes

1 V1.8.0, http://qiime.org/

2 v5.2.236, http://www.drive5.com/usearch/
