# Supplementary material for: Effects of Dietary Isomaltooligosaccharide Levels on the Gut Microbiota, Immune Function of Sows, and the Diarrhea Rate of Their Offspring
Source: Front Microbiol. 2021 Jan 8;11:588986. doi: 10.3389/fmicb.2020.588986 (PMC7820075; doi:10.3389/fmicb.2020.588986)

**Supplementary Infromation**

**Supplementary Table 1** Composition and nutrient levels of basal diets.

| Igredient (%) | Content | |
| --- | --- | --- |
|  | Late gestation | lactation |
| High quality northeast corn | 47.90 | 57.67 |
| Barley | 30.00 | 10.00 |
| Bran | 5.00 |  |
| Soybean meal, 43% | 12.10 | 22.60 |
| soybeans |  | 2.00 |
| Fish meal | 1.00 | 1.50 |
| soybean oil |  | 1.63 |
| Limestone | 1.00 | 1.40 |
| Ca HPO4 | 1.20 | 1.20 |
| Salt | 0.50 | 0.60 |
| Lys | 0.15 | 0.20 |
| DL-Met | 0.15 | 0.20 |
| Premix^a^ | 1.00 | 1.00 |
| Total | 100 | 100 |
| Nutrient composition^b^ | Late gestation | lactation |
| DE MJ·kg^-1^ | 3,150.00 | 3,400.00 |
| CP % | 13.50 | 17.50 |
| EE % | 2.50 | 4.80 |
| CF % | 3.24 | 2.82 |
| Ash % | 5.18 | 5.49 |
| Ca % | 0.95 | 1.00 |
| TP % | 0.60 | 0.62 |
| AP % | 0.35 | 0.45 |
| TLys % | 0.70 | 1.20 |

^a^ The premix provided the following per kg of diets: VA 10, 000 IU, VD_3_ 2, 100 IU, VE 45 IU, VK_3_ 2.0 mg, thiamine 3.0 mg, riboflavin 5.0 mg, VB_6_ 1.8 mg, VB_12_ 0.03 mg, cholinechloride 1, 000 mg, nicotinic acid 25.0 mg, pantothenic acid 15.0 mg, biotin 0.08 mg, folic acid 1.0 mg, Mn20.0mg, Zn 80.0 mg, Fe 80 mg, Cu 6.0 mg, I 0.15 mg, Se 0.25 mg.

^b^ Nutrient levels were calculated values

**Supplementary Table 2** Raw reads and selected effective sequences in each sample.

| Sample  Name | At Day 107 of Gestation | | | At Day 10 of Lactation | | |
| --- | --- | --- | --- | --- | --- | --- |
|  | sequences | OTUs | Phylum_OTUs | sequences | OTUs | Phylum_OTUs |
| CON-1 | 33247 | 5486 | 1381 | 35427 | 6204 | 1519 |
| CON-2 | 39638 | 6248 | 1546 | 42783 | 6576 | 1626 |
| CON-3 | 36625 | 6823 | 1730 | 37725 | 5931 | 1492 |
| CON-4 | 47692 | 7770 | 1956 | 50518 | 4482 | 1099 |
| CON-5 | 43067 | 7354 | 1843 | 45619 | 4809 | 1231 |
| IMO1-1 | 37736 | 6748 | 1717 | 35568 | 6512 | 1615 |
| IMO1-2 | 41104 | 7399 | 1882 | 39940 | 5724 | 1427 |
| IMO1-3 | 33644 | 6502 | 1621 | 36032 | 6022 | 1520 |
| IMO1-4 | 43354 | 7729 | 1933 | 31595 | 5563 | 1408 |
| IMO1-5 | 36323 | 6771 | 1690 | 33042 | 5526 | 1374 |
| IMO2-1 | 33643 | 5744 | 1473 | 40394 | 6280 | 1524 |
| IMO2-2 | 35858 | 6744 | 1700 | 33967 | 6052 | 1507 |
| IMO2-3 | 34761 | 6316 | 1574 | 45365 | 3881 | 942 |
| IMO2-4 | 35250 | 6043 | 1505 | 31233 | 5765 | 1486 |
| IMO2-5 | 27605 | 5488 | 1345 | 37069 | 6311 | 1605 |
| IMO3-1 | 45626 | 6056 | 1507 | 34990 | 6687 | 1649 |
| IMO3-2 | 49925 | 5702 | 1442 | 34390 | 5971 | 1494 |
| IMO3-3 | 28783 | 5327 | 1333 | 37049 | 6627 | 1657 |
| IMO3-4 | 27765 | 5628 | 1384 | 30734 | 5393 | 1351 |
| IMO3-5 | 32952 | 6152 | 1541 | 36563 | 6528 | 1618 |
| IMO4-1 | 35396 | 5864 | 1469 | 40339 | 7304 | 1820 |
| IMO4-2 | 31896 | 5420 | 1378 | 37998 | 6259 | 1559 |
| IMO4-3 | 49299 | 5488 | 1329 | 31865 | 6426 | 1635 |
| IMO4-4 | 43721 | 6281 | 1515 | 34712 | 6058 | 1510 |
| IMO4-5 | 38089 | 7000 | 1728 | 34831 | 6336 | 1595 |
| IMO5-1 | 30065 | 5664 | 1426 | 45726 | 5160 | 1303 |
| IMO5-2 | 29047 | 4772 | 1156 | 36817 | 6249 | 1570 |
| IMO5-3 | 34576 | 6030 | 1496 | 35501 | 6073 | 1475 |
| IMO5-4 | 27017 | 5389 | 1358 | 43103 | 5560 | 1372 |
| IMO5-5 | 30795 | 4750 | 1165 | 41241 | 4974 | 1209 |

CON, CON group; IMO1, 2.5 g/kg IMO group; IMO2, 5.0 g/kg IMO group; IMO3, 10.0 g/kg IMO group; IMO4, 20.0 g/kg IMO group; IMO5, 40.0 g/kg IMO group.

**Supplementary Fig. 1.** LEfSe analysis of the gut microbiota composition of sows at day 107 of gestation and at day 10 of lactation. (A) At day 107 of gestation, cladogram using LEfSe method indicating the phylogenetic distribution of gut microbiota in sows among all dietary treatment. Each successive circle represents a phylogenetic level. (B) At day 107 of gestation, histogram of the LDA scores reveals the most differentially abundant taxa among different dietary treatment. (C) At day 10 of lactation, cladogram using LEfSe method indicating the phylogenetic distribution of gut microbiota in sows among all dietary treatment. Each successive circle represents a phylogenetic level. (D) At day 10 of lactation, histogram of the LDA scores reveals the most differentially abundant taxa among different dietary treatment. GIMO1, 2.5 g/kg IMO group at day 107 of gestation; GIMO2, 5.0 g/kg IMO group at day 107 of gestation; GIMO3, 10.0 g/kg IMO group at day 107 of gestation, GIMO4, 20.0 g/kg IMO group at day 107 of gestation. LIMO2, 5.0 g/kg IMO group at day 10 of lactation; LIMO4, 20.0 g/kg IMO group at day 10 of lactation; LIMO5, 40.0 g/kg IMO group at day 10 of lactation.

B

A


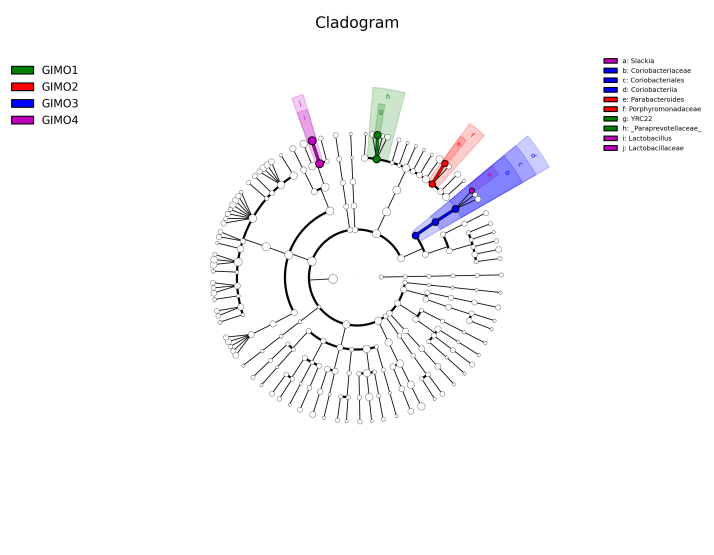

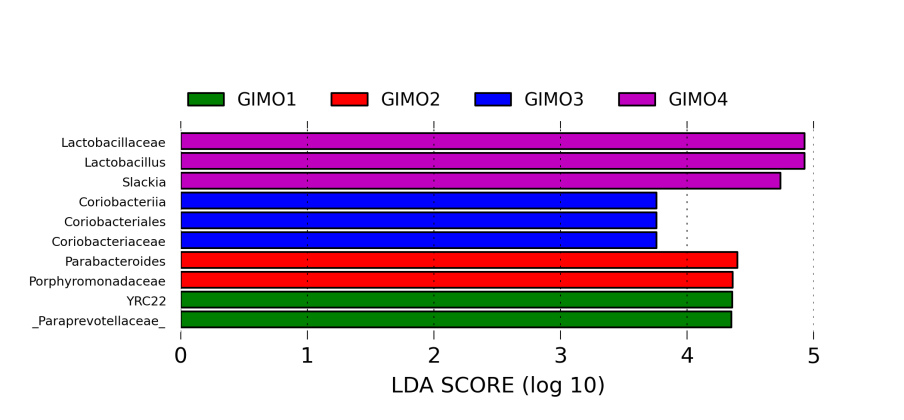


D

C


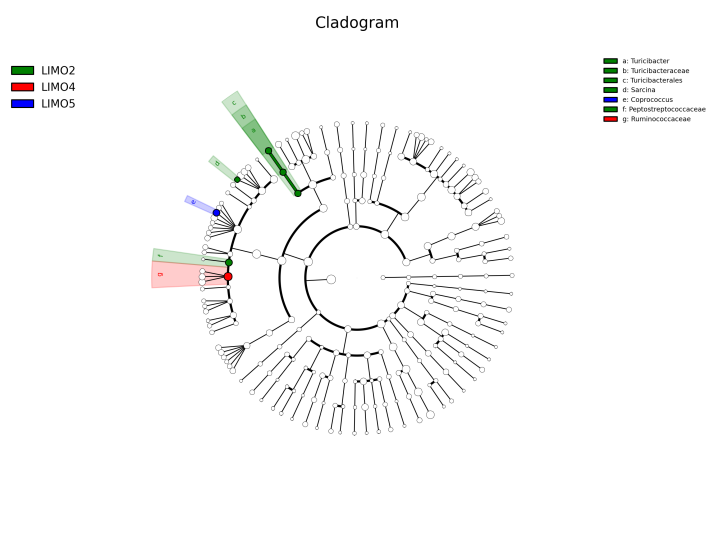

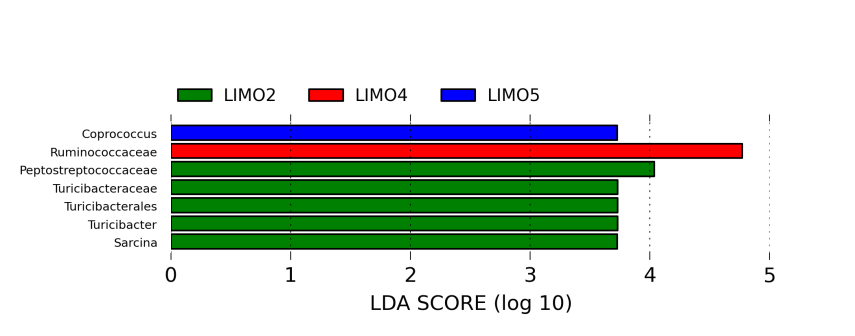

Supplement: Supplementary file 1 [file Data_Sheet_1.docx]
